# Correlations of Free Thyroid Hormones Measured by Tandem Mass Spectrometry and Immunoassay in Dogs

**DOI:** 10.3390/ani15050689

**Published:** 2025-02-27

**Authors:** Raffaella Sasso, Marcello Siniscalchi, Serenella d’Ingeo, Gianluca Ventriglia, Candida Bitetto, Angelo Quaranta

**Affiliations:** 1 Department of Veterinary Medicine, University of Bari Aldo Moro, 70121 Bari, Italy; marcello.siniscalchi@uniba.it (M.S.); serenella.dingeo@uniba.it (S.d.); gianluca.ventriglia@uniba.it (G.V.); angelo.quaranta@uniba.it (A.Q.); 2 ACV Triggiano S.R.L., Triggiano, 70019 Bari, Italy; c.bitetto@acvtriggiano.it

**Keywords:** dog, physiology, hypothyroidism, free thyroxine (FT4), thyroid-stimulating hormone (TSH), liquid chromatography–tandem mass spectrometry (LC-MS/MS), chemiluminescence immunoassay (CLEIA)

## Abstract

Thyroid function is commonly assessed in dogs through serum measurements of free thyroxine (FT4) and thyroid-stimulating hormone (TSH). The accurate measurement of thyroid hormones is critical. In particular, the measurement of free thyroxine (FT4) is important for the diagnosis and management of thyroid disorders. Most laboratories measure FT4 using direct analog immunoassay methods. The reliability of the results obtained using these methods has often been questioned. Fifty-nine dogs for which the veterinarian had requested the evaluation of thyroid hormones were included in this study. FT4 and TSH were measured using chemiluminescence enzyme immunoassay (CLEIA). Furthermore, FT4 was also measured by liquid chromatography–tandem mass spectrometry (LC-MS/MS) preceded by ultrafiltration. Comparing FT4 with the two different methods, the results demonstrate that LC-MS/MS, preceded by ultrafiltration, provides more accurate and specific FT4 measurements, particularly in dogs with altered thyroid hormone values. Although CLEIA is cost-effective and widely used for screening, LC-MS/MS is recommended for the more precise assessment of thyroid function.

## Simple Summary

The diagnosis of primary hypothyroidism in dogs is confirmed by a reduced serum total thyroxine (TT4) or free thyroxine (FT4) level associated with an elevated serum thyroid-stimulating hormone (TSH) level. To formulate a diagnosis, in addition to a correct evaluation of the medical history, direct physical examination and haemato-biochemical profile, an accurate measurement of the serum concentration of thyroid hormones is important. The aim of this study is to evaluate FT4 in suspected hypothyroid dogs by comparing two of the most accredited assay techniques in common veterinary laboratories: chemiluminescence enzyme immunoassay (CLEIA) and liquid chromatography–tandem mass spectrometry (LC-MS/MS) preceded by ultrafiltration. The results demonstrate that CLEIA, which is more advantageous and practical from an economic point of view, can be used for screening, while LC-MS/MS is recommended for a more precise evaluation of thyroid function in more complex clinical cases such as in dogs with altered thyroid hormone values. A significant and statistical positive correlation was observed between blood FT4 values measured by liquid chromatography–tandem mass spectrometry and chemiluminescence both in subjects with normal FT4 and TSH values and in the group of dogs that presented these altered values. Furthermore, a negative and significant association was observed between blood TSH and FT4 values measured with tandem mass spectrometry in the group of dogs with hormonal values attributable to hypothyroidism.

## 1. Introduction

Thyroid hormones (THs) play a crucial role in regulating various biological processes, including growth, metabolism, and development, in vertebrates. They also play a central role in the metabolism of lipids, carbohydrates, and proteins, as well as heart rate regulation, neural development, and the function of the cardiovascular, renal, reproductive and brain system. THs are synthesized in the thyroid gland and transported through the bloodstream to their target tissues [1].

The hypothalamus and pituitary gland are fundamental in regulating the production of thyroid hormones (THs). Neurons in the paraventricular nucleus within the hypothalamus secrete thyrotropin-releasing hormone (TRH), which stimulates the anterior pituitary gland to secrete thyrotropin/thyroid-stimulating hormone (TSH). Thyroid-stimulating hormone impacts on its receptors through thyroid follicular cells. This process comprises three stages: iodide uptake, iodide activation, and tyrosine iodination, which are essential for the proper synthesis and secretion of thyroid hormones (Ths) [2]. The negative feedback effect of thyroid hormones (in the free or unbound form) is the principal mechanism regulating TSH secretion. As the blood levels of T3 and T4 rise, the synthesis and release of TSH and TRH from the pituitary and hypothalamus are inhibited, helping to maintain stable thyroid hormone levels in the body. The hypothalamic–pituitary–thyroid (HPT) axis is essential for regulating the synthesis and secretion of Ths and interacts with other endocrine and physiological systems, forming an intricate regulatory network [3,4]. The metabolically active thyroid hormones are the iodothyronines: triiodothyronine (T3), which contains three iodines, and thyroxine (T4), which contains four iodines. Thyroxine is the main secretory product of the normal thyroid gland. However, triiodothyronine, which is three to five times more potent than T4, along with smaller amounts of inactive 3,3′,5′-L-triiodothyronine (reverse T3) and other deiodinated metabolites are also secreted [3]. Most of the thyroid hormones in the blood are bound to plasma proteins, such as thyroid hormone binding globulin (TBG), transthyretin (TTR, prealbumin), and albumin, only 0.3% of T3 and 0.03% of T4 are found in the free form and are not protein-bound [5]. Dogs have lower thyroid hormone binding to serum proteins than humans, resulting in lower total serum concentrations of T4, T3, higher free hormone concentrations, and more rapid clearance rates, and there is more T4 than T3 [6]. Only free hormones enter cells to produce a biological effect or a negative feedback effect on the pituitary and hypothalamus. T3 enters cells more rapidly and has a quicker onset of action than T4 [7,8]. However, most of T3 is formed from T4 in the target tissues by iodothyronine deiodinases type I and type II. T3 is physiologically active and regulates the expression of genes involved in the biological processes described above, by binding to thyroid hormone nuclear receptors [9,10].

Thyroid gland function in dogs is commonly evaluated through serum total thyroxine (TT4), free thyroxine (FT4) and thyrotropin (TSH) concentrations [11].

The techniques considered ‘gold standard’ for diagnosing hypothyroidism are thyroid scintigraphy and the rhTSH stimulation test [3]. However, these techniques are only available in certain specialized centers and can be expensive. Screening for the diagnosis of hypothyroidism and hyperthyroidism in veterinary medicine is represented by laboratory tests that measure hormone levels in the blood, in particular, the measurement of thyroxine (T4) and thyrotropin (TSH). Hypothyroidism can result from dysfunction at any point in the hypothalamic–pituitary–thyroid axis and may be acquired (most common) or congenital. In acquired canine hypothyroidism, most cases are attributed to primary hypothyroidism, caused by conditions like lymphocytic thyroiditis or idiopathic thyroid atrophy. Less frequently, primary hypothyroidism may result from bilateral thyroid neoplasia or the invasion of the thyroid by metastatic neoplasia [7].

Unfortunately, alteration in the serum concentrations of thyroid hormones is common in patients with systemic illnesses, even in the absence of thyroid disease.

This phenomenon is now commonly referred to as ‘Non-thyroidal illness syndrome (NTIS)’ describing the typical changes in thyroid-related hormone concentrations in the serum following any acute or chronic illness that is not caused by an intrinsic abnormality in thyroid function [12].

To reduce the risk of misdiagnosis, it is crucial to accurately measure the serum concentrations of TT4 and/or FT4 alongside the serum TSH concentration, taking into account the possibility that variations in the serum concentrations of TT4, FT4, and TSH can be influenced by factors other than hypothyroidism [11].

TT4 is vital for sustaining normal physiological functions, while FT4, in its unbound state in plasma, offers a more accurate measure of thyroid activity. TSH and FT4 are key tests for clinically assessing thyroid function. While TSH alone is insufficient for general screening, in association with FT4 testing, it is essential for diagnosing secondary thyroid disorders, subclinical thyroid dysfunction, and non-thyroidal illness syndromes. It is also crucial for monitoring treatment effectiveness and evaluating patient outcomes. The evaluation of FT4 (free thyroxine) instead of total T4 in the diagnosis of hypothyroidism and hyperthyroidism in veterinary medicine is generally considered more accurate. This is because FT4 represents the active fraction of the thyroid hormone, while TT4 can be influenced by various factors, such as the presence of bound proteins [5]. In dogs, among thyroid hormones, the measurement of FT4 concentration has the highest sensitivity, specificity, and accuracy for investigating hypothyroidism. The measurement of total T4 concentration has lower sensitivity, specificity, and accuracy. The TSH concentration has lower sensitivity and accuracy but the same specificity as measuring the FT4 concentration. When T4 (total or free) and TSH concentrations are assessed together, specificity is higher [11].

Table 1 presents the diagnostic sensitivity, specificity, and accuracy parameters for diagnosing canine hypothyroidism, based on the literature [11,13,14,15,16,17].

Sensitivity is defined as the proportion of hypothyroid dogs with abnormal serum hormone concentrations, while specificity refers to the proportion of euthyroid dogs with serum hormone concentrations within reference ranges. Accuracy is defined as the percentage of correct test results, regardless of whether the dogs are euthyroid or hypothyroid. This overview examines various thyroid tests used in veterinary medicine, including free thyroxine (FT4), total thyroxine (T4), thyroid-stimulating hormone (TSH), and combinations of these tests employed in serial and parallel testing. In serial testing, the results of both tests are combined, and the diagnosis is confirmed only if both tests are abnormal. This approach typically increases specificity but may reduce sensitivity. In contrast, parallel testing interprets both tests concurrently, where a positive result in either test is considered diagnostic. This generally increases sensitivity but may lower specificity. Among the different thyroid tests, the measurement of FT4 concentration demonstrates the highest sensitivity, specificity, and accuracy. The measurement of serum TT4 concentration also exhibits high sensitivity, specificity, and accuracy, though slightly lower than FT4. The measurement of TSH concentration shows higher specificity but lower sensitivity. When FT4 or TT4 and TSH are evaluated in series, specificity increases compared to measuring TT4 or TSH alone, but sensitivity decreases.

Accurately measuring hormones that circulate in low concentrations is a well-known challenge.

Clinical laboratories typically employ immunoassay (IA) methods or physical separation approaches for FT3 or FT4 testing. Immunoassays (RIA, ELISA, CLEIA), which rely on the specific interaction between antigens and antibodies, are performed in the presence of protein-bound T3 and T4, while the physical separation methods use techniques like equilibrium dialysis, ultrafiltration, or gel filtration to separate free hormone from protein-bound T3 (TT3) and T4 (TT4) [18].

Liquid chromatography–tandem mass spectrometry (LC-MS/MS) is a highly accurate technique with low limits of detection, and most importantly, it offers specificity for the analyte in question. LC-MS/MS is more specific than immunoassays when measuring thyroid hormones, making it an attractive method for thyroid hormone quantification [19].

Several studies have compared the results from LC-MS/MS, which is preceded by ultrafiltration, with those from LC-MS/MS that uses an equilibrium dialysis immunoassay to measure free thyroid hormones. The findings show that the results from the first method are very similar to those obtained using the gold standard method, which is Nichols equilibrium dialysis [18,20,21,22,23]. Furthermore, the ultrafiltration procedure can be performed in 30 min, while equilibrium dialysis takes 17 h [21,24]. Historically, a direct analog immunoassay for measuring free thyroid hormones has been criticized as suboptimal, primarily because it does not separate the hormones through ultrafiltration or dialysis before analysis [19]. Additionally, the presence of interfering thyroid antibodies or non-specific heterophile antibodies can affect the accuracy of chemiluminescence tests because these antibodies can bind to the reagents or components of the test, altering the reaction that is supposed to occur. This can lead to false negative results, making it difficult to obtain an accurate measurement of the substance being analyzed. Essentially, these antibodies can ’disturb’ the process, compromising the reliability of the test [25].

Our study aimed to compare the measurements of free thyroid hormones using immunoassay and LC-MS/MS, in order to investigate whether, even in the veterinary field, analyzing FT4 with LC-MS/MS instrumentation avoids interferents capable of altering the correct FT4 values or whether the chemiluminescence method, which in addition to being less expensive and easier to perform than LC-MS/MS, is not equally valid in dogs and capable of giving optimal FT4 values in a variety of clinical situations (altered values versus normal values). Furthermore, the relationship between FT4 and thyroid-stimulating hormone (TSH) was evaluated to obtain a physiological rationale on which to base the evaluation of FT4 assays.

## 2. Materials and Methods

This study was a retrospective study of samples received at laboratory ACV Triggiano from June 2021 to June 2023 for the measurement of FT4 and TSH. Fifty-nine samples from dogs sent to the laboratory for thyroid investigations were recruited for experimentation. Initially, the samples were seventy-two but there were dogs for which anamnestic data were completely missing, subjects for which the test was intended to monitor hypothyroidism (and were therefore under treatment), and subjects in which thyroid hormone values were collected from the same animal but not at the same time were excluded from the sample. This study is only a preliminary analysis.

The average age of the dogs was 87.4 months. Among them, 29 dogs (49%) were male, while 25 (42.5%) were female; the sex of 5 dogs (8.5%) was not specified. The dogs represented 18 different breeds, including 16 mixed breeds, 5 Labradors Retrievers, 5 Golden Retrievers, 3 Beagles, 3 Cavalier King Charles Spaniels, 2 Toy Poodles, 2 Cocker Spaniels, 1 of each of the following breeds: Rottweiler, Pitbull, Italian Volpin, Jack Russell, Maremma Abruzzo Shepherd, German Dachshund, English Bulldog, Lagotto, Greyhound, Boxer, Pomeranian Dog, Maltese, and Poodle.

Serum concentrations of free T4 were measured using LC-MS/MS with the Agilent 1200 series HPLC combined with the Agilent 6490 triple quadrupole mass spectrometer equipped with ESI Jet Stream source (Santa Clara, CA, USA). These measurements were then compared to values obtained from a direct veterinary FT4 Chemiluminescence Enzyme Immunoassay (CLEIA) performed on the IMMULITE^®^ 2000 XPi Systems by Siemens Healthcare, Erlangen, Germany.

The serum TSH concentration was measured using the chemiluminescent immunoenzymatic technique (Immulite 2000^®^ XPi Systems by Siemens Healthcare Erlangen, Germany)). We collected samples from fifty-nine outpatients who were undergoing routine medical care and were suspected to have hypothyroidism.

### 2.1. Immunoassay

Chemiluminescence enzyme immunoassay is a technique that involves an immune reaction between the reagent antibodies and the analyte in the sample. In this process, the alkaline phosphatase-labeled reagent component (referred to as the conjugate) binds to a bead inside the reaction cuvette. Subsequently, a luminogenic substrate is introduced into the reaction cuvette, and the light generated during the luminogenic reaction is measured. During the reaction, the substrate (an adamantyl dioxetane phosphate) undergoes dephosphorylation by alkaline phosphatase. The amount of emitted light is directly proportional to the bound alkaline phosphatase.

The canine reference intervals for FT4 measured with chemiluminescence are 10.5–41.0 pmol/L (the reference values for hormonal determinations were those established by the laboratory in which the analyzes were carried out).

The reference values we used for TSH (0.03 to 0.40 ng/mL) are the standard ones used for routine analysis performed by the laboratory.

Sample preparation: to obtain the sample, blood is centrifuged, and the serum is collected.

Equipment used: the Immulite^®^ 2000 XPi is a continuous random-access system designed for chemiluminescence immunoassays. This system utilizes serum, plasma, or urine samples for in vitro diagnostic tests and seamlessly integrates with RealTime SolutionsSM and VersaCell^®^ systems.

Instrumental analysis: IMMULITE^®^ 2000 Sistem Veterinary FreeT4 is a competitive, chemiluminescent, solid phase analog immunoassay. The solid phase (sphere) is coated with a mouse anti-T4 monoclonal antibody. The liquid phase consists of alkaline phosphatase (bovine calf intestine) conjugated to T4. The animal sample and reagent are incubated together with the coated sphere for 30 min. During this period, free T4 competes with T4 conjugated with the enzyme in the reagent for a limited number of antibody binding sites on the sphere. The unbound patient sample and the conjugate enzyme are then removed by centrifuge washing. Finally, a chemiluminescent substrate is added to the reaction tube containing the sphere and the signal is generated in proportion to the bound enzyme. Regarding the Immulite^®^ 2000 Veterinary FT4 assay by Siemens Healthcare Diagnostics Ltd., as reported by the manufacturer, this assay has an analytical sensitivity of 0.51 pmol/L and a lower detection limit of 1.29 pmol/L. Despite a reported functional sensitivity of 3.2 pmol/L, the assay’s calibration range extends from 3.86 to 77.2 pmol/L. The manufacturer reports intra-assay and inter-assay coefficient of variations (CVs) of 10.8%, 7.6%, 5.9%, 5.0%, and 3.5%, and 11.9%, 8.3%, 6.5%, 5.9%, and 4.3% for FT4 concentrations of 7.6, 13.8, 24.2, 40.5, and 75.5 pmol/L, respectively. The reference interval provided by the manufacturer is 7.7 to 47.6 pmol/L. All samples, adjustors, and bi-level quality control samples were processed according to the manufacturer’s instructions. For TSH intra-assay CVs were 5%, 4%, and 3.8% at TSH concentrations of 0.2, 0.5, and 2.6 ng/mL^−1^, respectively. The inter-assay CVs were 6.3% and 8.2% at TSH concentrations of 0.16 and 2.8 ng/mL^−1^, respectively. The sensitivity of the assay was 0.03 ng/mL^−1^ [26].

As reported by the manufacturer, the intraassay CVs for TSH were 5%, 4% 3.1%, and 3.8% at TSH concentrations of 0.2, 0.5, 1.6, and 2.6 ng/mL, respectively. The intraassay CVs were 6.3%, 7.4%, and 8.2% at TSH concentrations of 0.16, 0.27, and 2.8 ng/mL, respectively. The sensitivity of the assay was 0.03 ng/mL, and indeed, the lowest detectable concentration of TSH was 0.03 ng/mL [27].

### 2.2. LC-MS/MS

Liquid chromatography–tandem mass spectrometry is a technique used to identify the analytes in a sample based on the retention time and mass-to-charge ratio of parent and fragmentation ions as they pass through a vacuum.

The process begins with the extraction of molecules, peptides, and small proteins with the Agilent 1200 Series HPLC, a high-performance liquid chromatography (HPLC) system for the separation and detection of organic compounds in mixtures. Samples are dissolved in an HPLC-compatible solvent and placed into autosampler vials in the autosampler, from which they are injected into a solvent stream called the “mobile phase” (water/methanol or water/acetonitrile for reversed-phase HPLC). The injected sample passes through a reversed-phase C18 column (the “stationary phase”) where analytes are separated by competitive interactions between the polar mobile phase and the nonpolar stationary phase. Smaller, more polar/acidic/basic compounds will tend to elute from the column before larger, less polar/acidic/basic compounds, resulting in spatial separation of analyte molecules in the mobile phase stream. The separate analyses are then detected by a variable wavelength UV detector. The sample is then ionized in the ion source, also called an ionization chambre. A mass spectrometer works by converting individual molecules into ions, which it then analyzes for relative abundance. In the ionization chamber of a mass spectrometer, each individual molecule is ionized to form a molecular ion that has one fewer electron than the parent molecule. The molecular ions (or “radical cations”) are then fragmented into smaller ions, which are further fragmented. From a single complex sample, a mass spectrometer generates many ions. The ions are then accelerated in an electromagnetic field and separated based on their mass-to-charge ratio (*m*/*z*).

Subsequently, the ions are analyzed by a mass analyzer (mass filter) that controls their movement towards the detector, where they are converted into actual signals.

The quantification of analytes is achieved by determining the ratio of the analyte to an isotopically labeled internal standard (deuterated or carbon-13).

Tandem mass spectrometry (MS/MS) allows for the selection of the ion of interest and the generation of characteristic fragments through one or more collisions (CAD, collision-activated dissociation). In the collision process, a fraction of the kinetic energy of the ion is converted into internal energy, causing it to dissociate into various fragments; the extent of fragmentation depends on the total internal energy content of the excited ion. There are two main categories of instruments that enable MS/MS experiments. Space tandem spectrometers, the first designed for the sequential assembly of two sector analyzers, followed by two quadrupoles, time-of-flight (TOF), or their combinations. Time tandem spectrometers, in which ion selection and dissociation occur within the same space but at successive times, and other instruments of this type include the quadrupole ion trap and the cyclotron resonance ion trap. The tandem mass spectrometer we use is the triple quadrupole.

The canine reference intervals for FT4 measured with LC-MS/MS are 12.8–47.3 pmol/L (the reference values for hormonal determinations were those established by the laboratory in which the analyzes were carried out).

Sample preparation: to obtain the sample, blood is collected in tubes without separating gel and is centrifuged, and the serum is collected. Sample preparation involves the separation of the free fraction of thyroid hormones from the conjugated fraction using the Centrifree^®^ ultrafiltration device with Ultracel^®^ (Darmstadt, Germania) regenerated cellulose membrane, and by liquid–liquid extraction (HPLC).

Instrumentation used: the equipment used for LC-MS/MS includes the Agilent 1200 series HPLC in combination with the Agilent 6490 triple quadrupole mass spectrometer, featuring an ESI Jet Stream source (Agilent Technologies, Santa Clara, CA, USA).

Equipment used: chromatographic separation (HPLC) is performed on a Zorbax SB-C18 Rapid Resolution HT 2.1 × 50 mm, 1.8 µm chromatographic column using a gradient elution method with mobile phases A (0.1% formic acid in water) and B (methanol). The initial B percentage is 50%, and maintained for 0.5 min, followed by a linear increase to 85% B at 2.5 min and further increase to 98% B at 2.6 min. This percentage remains constant until 4 min and then returns to the initial condition at 4.25 min, with a reconditioning time of 1.75 min.

Detection is carried out by chemical ionization in electrospray in positive mode employing MRM (multiple reaction monitoring) for the following transitions: Precursor ion 777.5; product ions 731.5 (quantifier), 323.8 (first qualifier), and 196.8 (second qualifier).

With ultrafiltration plus the LC/MS/MS assay, the between-day and within-day precision results indicate that all tested concentrations yielded coefficient of variations (CVs) of less than 7.1%. Specifically, Soldin showed CVs ranging from 4.1% to 6.6%, while Hernández had CVs below 7.2%. The lower limit of detection, defined as a reading greater than +3 standard deviations (S.D.) over baseline noise, was 2.5 pg/mL [21,28].

### 2.3. Data Analysis

The statistical analysis was performed using SPSS software version 22 (IBM, Armonk, NY, USA). Data distribution was tested using the Shapiro–Wilk test. According to data distribution, Spearman correlations were performed to study possible association between FT4 and TSH measured through liquid chromatography–tandem mass spectrometry and Chemiluminescence Enzyme Immunoassay in both altered values versus normal values dogs. Spearman coefficient (r) was calculated as a Pearson correlation coefficient but using the ranks of the values of each of the 2 variables analyzed instead of their actual values. Results were considered statistically significant for *p* < 0.05.

Furthermore, Bland and Altman plots were used in order to quantify the difference between LC-MS/MS and CLEIA FT4 measurements in “normal values” and “altered values” using a graphical method.

Generalized linear mixed model (GLMM)) analysis was carried out to assess the influence of “sex”, “age”(the sample population has divided into three categories: 0–24 months “juvenile” *n* = 1; 24–120 months “adult” *n* = 36; >120 months “old” *n* = 7) and “breed” (mongrels *n* = 17 and purebreds *n* = 33) on the test variables “FT4 in liquid chromatography–tandem mass spectrometry”, “FT4 in chemiluminescence immunoassay” and “TSH in chemiluminescence immunoassay”, with “subjects” as a random variable. Bayesian information criterion (BIC) was used for selecting and comparing models based on the −2log likelihood. To detect the differences between different groups, Fisher’s least significant difference (LSD) pairwise comparisons were performed. Fifteen and nine data were missing from the database, respectively, for the “sex” and “breed” variables.

## 3. Results

**Figure 1:**
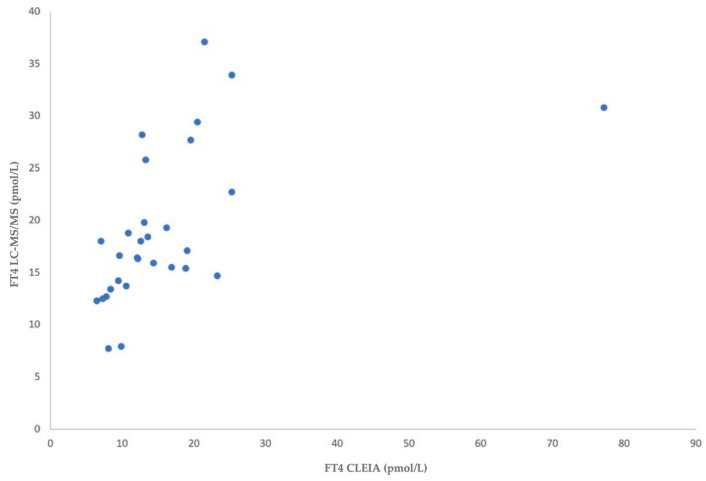
Associations for FT4 in liquid chromatography–tandem mass spectrometry and FT4 in chemiluminescence immunoassay in dogs with normal values.

**Figure 2:**
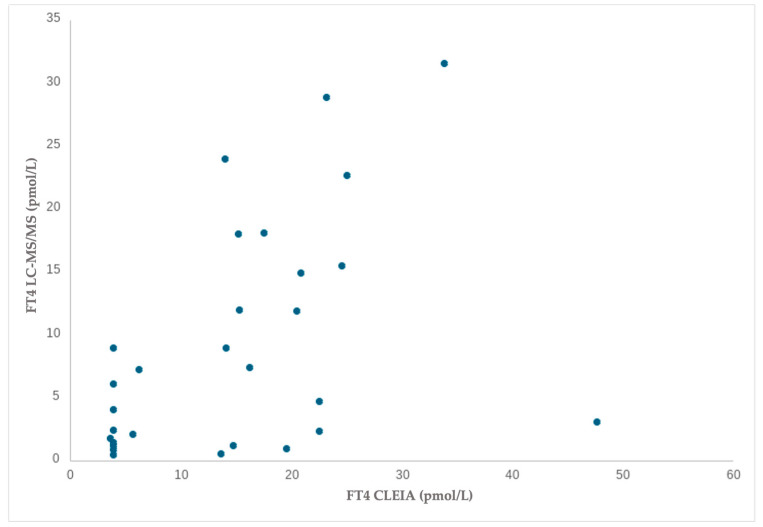
Associations for FT4 in liquid chromatography–tandem mass spectrometry and FT4 in chemiluminescence immunoassay in dogs with altered values.

Of the 59 tested dogs, 30 samples (50.8%) had TSH between 0.04 and 4.00 mIU/L and FT4 within the reference range, while twenty-nine (49.2%) had TSH > 4.00 mIU/L and FT4 below the reference values. A Spearman’s correlation comparing the liquid chromatography–tandem mass spectrometry and chemiluminescence immunoassay demonstrated a moderate or strong positive and significant association for FT4 blood values in both altered values versus normal values dogs: ‘‘normal values’’ (r(30) = 0.688, *p* =0.000) (Figure 1); ‘‘altered values’’ (r(29) = 0.549, *p* = 0.002) (Figure 2).

In this study, we used the Spearman’s rank correlation coefficient (r) to assess the strength and direction of the monotonic relationship between clinical variables utilized by Schober et al. [29]. The r-value indicates the degree of association between two variables, with values ranging from 0.00 (very weak correlation) to 1.0 (perfect correlation). In general, a positive r-value reflects a positive relationship, while a negative value indicates a negative one. The closer the r-value is to 1 or −1, the stronger the relationship.

The results for the “normal values” group show a strong positive correlation (r = 0.688, *p* < 0.001), indicating a significant relationship between the variables. The “altered values” group shows a moderate positive correlation (r = 0.549, *p* = 0.002), suggesting a weaker but still meaningful association. Both correlations are statistically significant, with the relationship being stronger in the normal values group.

**Figure 3:**
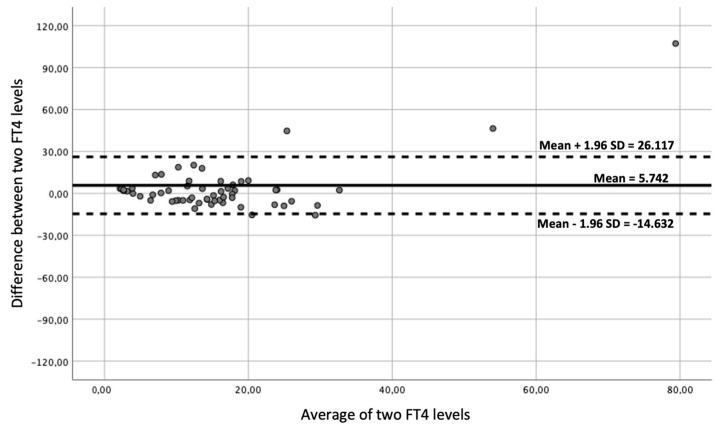
Agreement between FT4 measurements in liquid chromatography–tandem mass spectrometry and in chemiluminescence immunoassay in dogs with normal values (Bland–Altman plot).

**Figure 4:**
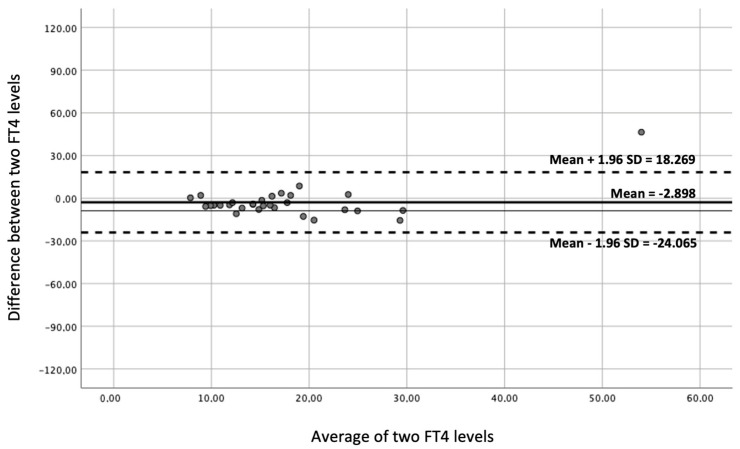
Agreement between FT4 measurements in liquid chromatography–tandem mass spectrometry and in chemiluminescence immunoassay in dogs with altered values (Bland–Altman plot).

Bland–Altman plots comparisons between LC-MS/MS and CLEIA FT4 measurements in “normal values” and “altered values” were shown in Figure 3 and Figure 4, respectively.

**Figure 5:**
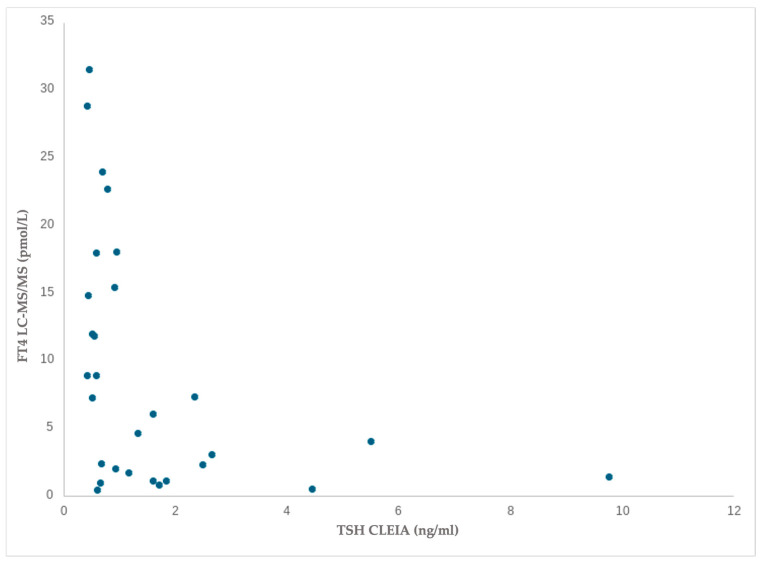
Associations for FT4 in liquid chromatography–tandem mass spectrometry and TSH in chemiluminescence immunoassay in dogs with altered values.

With regard to TSH blood values, a moderate negative and significant association was observed only with respect to FT4 measured with tandem mass spectrometry in dogs with altered values: (r(29)= −0.565, *p* = 0.001) (Figure 5).

No other statistically significant correlations were found between TSH and Ft4: *p* > 0.05 for all comparisons.

The analysis revealed a significant effect of the “sex” variable on the TSH values; specifically, TSH levels in chemiluminescence immunoassay were higher in female compared to male dogs (males: 0.59 ± 0.11 (m ± sem); females 1.12 ± 0.42 (m ± sem); (F(1,36) = 4.449, *p* = 0.042)). No other significant differences with respect to “sex”, ”age”, and ”breed” were observed (TSH-CLEIA: “age” (F(2,36) = 0.329, *p* = 0.722); “breed” (F(2,36) = 2.907; *p* = 0.068); FT4-CLEIA: “sex” (F(1,36) = 0.026, *p* = 0.874, “age” (F(2,36) = 0.151, *p* = 0.860); “breed” (F(2,36) = 0.494; *p* = 0.614); F4-LC-MS/MS: “sex” (F(1,36) = 0.001, *p* = 0.971), “age” (F(2,36) = 2.510, *p* = 0.917); “breed” (F(2,36) = 0.087; *p* = 0.917).

## 4. Discussion

Radioimmunoassay (RIA), the first method developed for detecting FT4, utilizes a competitive inhibition reaction where radiolabeled and non-labeled antigens compete for binding with antibodies to identify the target antigen [30]. Chemiluminescent immunoassay (CLEIA), which utilizes the generation of photons/light as a product of a chemical reaction, has become an attractive alternative to substitute RIA because it is safe for humans and the environment and easy to use [31].

The more classical ELISA, instead, is a solid-phase enzyme-based immunoassay technique, is convenient and simple to perform but has lower sensitivity and longer reaction times. As a result, it is less frequently used in clinical practice and is gradually being replaced by CLEIA [32].

Siemens Healthineers has developed and marketed several series of IMMULITE^®^ systems using CLEIA technology including IMMULITE^®^ 1000, 2000, and 2000 XPi to the measure concentrations of different hormones. CLEIA integrates immunoassay techniques with chemiluminescent systems and is currently the fastest-growing and most widely adopted immunoassay method [33].

The procedure with IMMULITE 2000 Veterinary FT4 is a direct or single test, in the sense that its results are not calculated as a function of total T4 but interpolated by a standard curve calibrated in terms of free T4 concentrations [34]. This differs from the classical methods of equilibrium dialysis and also from the so-called free T4 index determinations. It requires neither a pre-incubation phase nor a preliminary isolation of the free fraction by dialysis or column chromatography, and for this reason, it is a rather quick test. The test uses several features to preserve the balance between free and protein bound T4 and to measure the unbound fraction. First, the analogue T4 has an undetectable binding affinity for TBG. Second, the antibody specific to the test binds T4 as much as albumin binds T4 and thus prevents the elimination of the hormone from thyroid-binding proteins.

No immunoassay method is free from nonspecific antibody interference. When analyzing FT4 results, it is necessary to consider the patient’s clinical symptoms. It should be assessed whether the laboratory results are correct using other measures, and when discrepancies occur between test parameters or between test results and clinical symptoms, potential interfering factors, such as medication use or the presence of autoantibodies, should be evaluated to prevent misdiagnosis and inappropriate treatment [5].

LC-MS/MS technology is considered a “gold standard” for the precise detection of small molecules and is therefore globally considered as one of the most reliable techniques both from a qualitative and a quantitative point of view [35]. It integrates the exceptional separation capability of liquid chromatography with the high sensitivity and specificity of mass spectrometry. However, the use of LC-MS/MS in FT4 testing remains limited due to the high cost of the equipment and the time- and labor-intensive nature of the procedure.

Furthermore, this technique requires high analyte purity; therefore, the FT4 assay involves sample preparation methods, so that the sample is purified to minimize the presence of interfering substances. Consequently, sample pretreatment is crucial to eliminate macromolecules, lipids, and other impurities from biological matrices. The free hormone hypothesis postulates that only the unbound fraction (the free fraction) of hormones circulating in the blood bound to their carrier proteins can enter cells and exert its biological effects [36]. Physiological effects are therefore dependent on the 0.03% of T4 circulating in its free form. The concentration of the free fraction is mainly governed by the levels and binding affinity of the binding proteins, maintaining a dynamic equilibrium between the free and bound states [37]. Therefore, it has been widely accepted that measuring free T4 levels may be a more important element in correctly assessing thyroid function, because total T4 levels (protein-bound T4 and unbound T4) can be affected by fluctuating carrier protein concentrations or binding capacities. Due to the dynamic equilibrium between free and bound forms, the most crucial step in sample preparation for FT4 testing is isolating the true free hormone fraction.

Equilibrium dialysis (ED) and ultrafiltration (UF) are the two primary methods for separating FT4 [38]. ED is regarded as the “gold standard” for free hormones separation. In this method, the solution to be dialyzed is placed on one side of a dialysis membrane, while a specific buffer is placed on the other. This setup allows small molecules to equilibrate between the sample and the dialysis fluid. For hormones, equilibrium is established on the serum side of the membrane, mirroring the original serum’s balance between bound and free hormones. ED separates the serum or plasma into two fractions: a protein-bound fraction in equilibrium with an unbound fraction and a non-protein-bound fraction. This results in an excess of bound hormones compared to free hormones [39]. UF is a pressure-driven membrane filtration process that separates small solvent and solute molecules from a solution containing larger molecules by applying a pressure gradient across a semi-permeable membrane.

UF is comparatively more convenient and faster, reducing filtration time. However, large molecules can accumulate to form a gel-like layer, increasing the transmembrane resistance, making regular cleaning and membrane replacement essential. Additionally, the mechanical nature of the filtration process increases the risk of protein loss during UF [40]. The direct measurement of FT4 (but also FT3) by equilibrium dialysis or ultrafiltration essentially serves as a reference method for standardizing immunological procedures mainly used in routine diagnostics.

ED applications combined with mass spectrometry have also been described, which have been proposed as a human reference method, but also ED combined with immunoenzymatic technique.

Soldin et al. developed an analytical method [21] in human serum/plasma using ultrafiltration (UF) in combination with LC-MS/MS [41]. While the ED step is time-consuming and technically demanding, the UF step has the advantage of rapid separation (about 30 min), but the accuracy and robustness of the UF step in laboratories have not been fully validated [42]. Many studies have reported that LC-MS/MS for FT4 allows much more precise measurements than those obtained by immunoassays, especially in pathological situations [9,21,35,43].

LC-MS/MS ionizes the substance to be measured and then separates the ions based on their mass/charge ratio (*m*/*z*). Combining the superior separation capability of chromatographic techniques with the exceptional selectivity, specificity, and sensitivity of mass spectrometry, this approach eliminates the nonspecific interferences commonly encountered in immunoassays. In our study, the results measured by chemiluminescence and by LC–MS/MS preceded by UF did not give particularly divergent results. The results obtained from the study on the 59 dogs tested offer interesting insights into the relationship between TSH and FT4 values. Spearman correlation analysis revealed a positive correlation between FT4 values measured by liquid chromatography–tandem mass spectrometry and those obtained by chemiluminescence immunoassay, both in dogs with normal and abnormal values. While the correlation is stronger in the normal values group, both sets of results are statistically significant, highlighting the importance of considering the relationship between FT4 and clinical measures in dogs, even when values are altered. This result is particularly encouraging, as it suggests that both measurement methods can provide consistent information on thyroid function. The FT4 values obtained with the two methods are statistically correlated, although the values obtained, in absolute terms, are not exactly the same. In addition, Wilcoxon’s signed-ranks test revealed that there was no significant statistical difference between the two different assay techniques (R = 860.500, N = 59 individuals, Z =−0.185, *p* = 0.853).

However, it is noteworthy that, for TSH values, a significant negative correlation with FT4 was observed only in dogs with altered values, as measured by liquid chromatography–tandem mass spectrometry (*p* = 0.001).

In subjects with altered hormonal values, the FT4 value measured by mass spectrometry is lower than that measured by chemiluminescence, and therefore, more consistent with the increase in TSH which exerts a negative feedback mechanism on the production of thyroid hormones.

Regarding TSH, thirty samples (50.8%) had a TSH between 0.04 and 4.00 mIU/L and twenty-nine (49.2%) had TSH > 4.00 mIU/L. It is worth noting that, in dogs, measuring the TSH concentration alone tends to be less specific and accurate than evaluating it together with FT4. In fact, about 30% of hypothyroid dogs may have TSH levels that fall within the reference range. Therefore, it is always beneficial to assess FT4/TT4 levels in conjunction with TSH for a more comprehensive evaluation [11].

With the mass spectrometry method FT4 and TSH are significantly inversely correlated in dogs with altered values of thyroid hormones, while in chemiluminescence, there is no correlation in dogs with altered values of thyroid hormones. This suggests that this method more accurately reflects the true FT4 concentrations. One possible reason is that the FT4 concentration in chemiluminescence is influenced by the concentration of carrier proteins and therefore may not be a true reflection of the free hormone concentration [22]. The ultrafiltration system used in mass spectrometry to measure FT4 effectively removes carrier proteins [44] and therefore the amount of bound T4 from the sample, thus replacing the preventive step of dialysis equilibration.

The poor correlation between FT4 measured by chemiluminescence and TSH shows that immunoassays to measure FT4 are more limited in subjects with values attributable to hypothyroidism.

Our study compared dogs with normal FT4 values to those with altered values. The statistical analysis revealed that in dogs with normal thyroid values, the FT4 levels obtained using the chemiluminescence method are like those measured by mass spectrometry. However, in dogs with thyroid hormone levels indicative of hypothyroidism, the results obtained through mass spectrometry differ significantly from those obtained via chemiluminescence.

In veterinary medicine, there is a lack of sufficient data in the literature regarding the dosage of FT4. A 2018 study conducted on dogs highlighted significant discrepancies between FT4 levels measured using two different methods. The authors suggested—though without being able to prove—that the discrepancy may have been due to the CLEIA test’s mechanism for measuring serum FT4. In this test, enzymatically labeled T4 competes for a limited number of binding sites. If anti-thyroid autoantibodies are present, which could interfere by competing with FT4, potentially affecting the results [9].

The analysis demonstrated a significant impact of the ’sex’ variable on TSH levels. Specifically, TSH values measured through the chemiluminescence immunoassay were found to be higher in female dogs compared to their male counterparts.

These data are interesting as it appears in the literature that women tend to have higher levels of TSH (thyroid-stimulating hormone) compared to men. This may be due to various factors, including hormonal differences and the fact that women are more susceptible to thyroid disorders, such as hypothyroidism [23,45,46].

## 5. Conclusions

The LC-MS/MS method can involve higher costs, both for the purchase and maintenance of the equipment and for the purchase of reagents. Additionally, it requires highly specialized personnel for the operation and interpretation of the results. However, it offers significant advantages in terms of precision and reliability, which could reduce the need for repeated tests. On the other hand, chemiluminescence is a more accessible and faster solution, although it may not always guarantee the same quality of results. It is important to note that the cost difference can vary significantly depending on the region, laboratory, and technologies used. In Italy, for example, the cost of FT4 testing via chemiluminescence is on average half that of the same test performed using the LC-MS/MS method.

Immunoassays are the primary method for clinical FT4 testing, valued for their simplicity, cost-effectiveness, and high level of automation. However, numerous factors can interfere with test results. Variations in antibodies, test protocols, and instrument calibration can cause inconsistencies in immunoassay outcomes. When the discrepancies in test results are suspected, thyroid function should be evaluated along with clinical indications and compared with more accurate test methods. Thus, in conclusion, considering their high automation, user-friendliness, and low cost, it is recommended to use chemiluminescence for FT4 testing in the case of screening, but, since clinical guidelines recommend using FT4 and TSH as a tool for the evaluation of thyroid diseases, it is advisable to use the LC-MS/MS method in case of hypothyroidism in dogs, which is more sensitive and specific and can compensate for the limitations of immunoassays due to various interferences, which may mislead clinical decisions about treatment.

## Figures and Tables

**Table 1:** The diagnostic sensitivity, specificity, and accuracy parameters for diagnosing canine hypothyroidism.

Parameter	Sensitivity	Specificity	Accuracy
Low TT4	89–98%	73–82%	78–85%
Low FT4	80–98%	78–94%	86–95%
High TSH	58–87%	82–93%	84–90%
Low TT4 and high TSH	67–87%	92–98%	82–88%
Low FT4 and high TSH	74–80%	97–98%	86–88%

## Data Availability

The datasets generated and/or analysed during the current study are available from the corresponding author upon reasonable request.

## Abbreviations

The following abbreviations are used in this manuscript:

CLEIA	Chemiluminescence enzyme immunoassay
LC-MS/MS	Liquid chromatography–tandem mass spectrometry
T4	Thyroxine
FT4	Free thyroxine
TSH	Thyrotropin/thyroid-stimulating hormone
TRH	Thyrotropin-releasing hormone
TT4	Total thyroxine
T3	Triiodothyronine
FT3	Free triiodothyronine
TT3	Total triiodothyronine
THs	Thyroid hormones
HPT	Hypothalamic–pituitary–thyroid
RIA	Radio-immunoassay
HPLC	High-performance liquid chromatography
ED	Equilibrium dialysis
UF	Ultrafiltration
NTIS	Non-thyroidal illness syndrome
